# Environmental asbestos exposure and mesothelioma cases in Bari, Apulia region, southern Italy: a national interest site for land reclamation

**DOI:** 10.1007/s11356-018-1618-x

**Published:** 2018-03-25

**Authors:** Luigi Vimercati, Domenica Cavone, Piero Lovreglio, Luigi De Maria, Antonio Caputi, Giovanni Maria Ferri, Gabriella Serio

**Affiliations:** 1 Interdisciplinary Department of Medicine (DIM), Unit of Occupational Medicine, University Aldo Moro of Bari Medical School, 11 G. Cesare Square, 70124 Bari, Italy; 2 Department of Emergency and Organ Transplantation (DETO), Pathology Division, University Aldo Moro of Bari Medical School, 11 G. Cesare Square, 70124 Bari, Italy

**Keywords:** Asbestos, Environmental-residential-neighborhood exposure, Mesothelioma, Contaminated town, Military barracks, Bari, Apulia. Italy, Mesothelioma registry, Public health

## Abstract

Asbestos is an environmental carcinogen, and asbestos-related diseases are a global-scale public health issue. We report three cases (one male and two females) of pleural malignant mesothelioma (PMM) caused by environmental asbestos exposure reported by the Apulia Regional Operating Centre (COR) to the National Mesothelioma Registry (ReNaM). The patients revealed no history of asbestos exposure even after detailed assessment. The environmental (neighborhood) asbestos exposure for each of the three cases was due to both the residential history of the subjects and their workplace, close to a military barracks, at a distance of between 45 and 100 m. Moreover, in addition to this new source of pollution, an asbestos cement factory was located in the urban area of Bari municipality, in the Apulia region, southern Italy. Environmental-residential/neighborhood asbestos exposure in the city of Bari, a contaminated area classified as a site of national concern for land reclamation, is discussed also with reference to the military barracks.

## Introduction

Malignant mesothelioma (MM) is a rare, lethal malignancy caused primarily by occupational or environmental asbestos exposure (Bourdès et al. 2000; Delgermaa et al. 2011; Lacourt et al. 2014; Røe and Stella 2015). MM generally has a poor prognosis, and cases are typically diagnosed at an advanced stage of the disease. Recently, with therapeutic advancements, the survival has improved (Reid et al. 2014; Faig et al. 2015). There is no evidence of a minimum asbestos exposure threshold to delineate an absence of risk (Hillerdal 1999; Goldberg and Luce 2009).

Not all people exposed to asbestos develop mesothelioma; thus, an underlying susceptibility to asbestos-related carcinogenesis may exist (Dogan et al. 2006). Cytogenetic studies have shown that MM has highly complex and variable chromosomal aberrations and asbestos exposure has been reported to cause genetic alterations at the chromosomal level in MM (Borczuk et al. 2016).

Recent studies have confirmed a significant MM risk due to asbestos environmental exposure (EE) (Baumann and Carbone 2016; Liu et al. 2017). Many studies have demonstrated an increased risk in the general population associated with a low environmental-type asbestos exposure (Magnani et al. 1995; Bourdès et al. 2000; Boffetta and Stayner 2006; Kurumatani and Kumagai 2008; Reid et al. 2008; Goldberg and Luce 2009; Marsh et al. 2017). EE can originate from pollution by industrial sites or mines, from the presence of asbestos in buildings (asbestos in a place), and from natural contamination of the soil, allowing for exposure that can begin at birth (Pasetto et al. 2005).

In Italy, Casale Monferrato, where an asbestos cement factory (Eternit) was located, is a dramatic example of asbestos pollution risk for people living in a contaminated area.

A recent study (Ferrante et al. 2016) provides strong evidence of an association between pleural mesothelioma and non-occupational exposures to asbestos. An approximately twofold increase in risk was observed for having lived with a family member who worked in the Eternit asbestos cement plant (OR = 2.4, 95% CI 1.3 to 4.4), or having been exposed from domestic or environmental sources (OR = 2.0, 95% CI 1.2 to 3.2).

The area of Bari municipality, Apulia region of southern Italy, has been defined as a contaminated site (CS) of national priority for remediation because of diffuse environmental contamination caused by an asbestos cement factory (Fibronit). Bari municipality was included in the Italian national priority list of contaminated sites in 2000 (law 388/2000). The former asbestos cement plant (Fibronit) operated from 1933 until 1985 and employed approximately 417 workers. (Coviello et al. 2002; Musti et al. 2009). Chrysotile (80%), crocidolite (15%), and amosite (5%) were used in the plant. The contaminated site, as defined by the law, includes an area of approximately 150,000 m^2^ (Ministerial Decree 468/01). Currently, definitive environmental remediation action is in progress and definitive work to increase safety started in October 2016. The environmental risk of the Fibronit company was confirmed in 2009. A spatial case-control study of the environmental impact of asbestos fibers spread by the Fibronit plant, analyzing data from the database of the Apulia Regional Operating Centre (COR) of the National Mesothelioma Registry (ReNaM), showed an MM odds ratio of 5.29 (95% CI 1.18–23.74) for people living close to the factory and residents within a range of up to 500 m from the factory. A low risk was found with increasing distance from the factory, which was the major urban source of asbestos pollution (Musti et al. 2009).

**Fig. 1:**
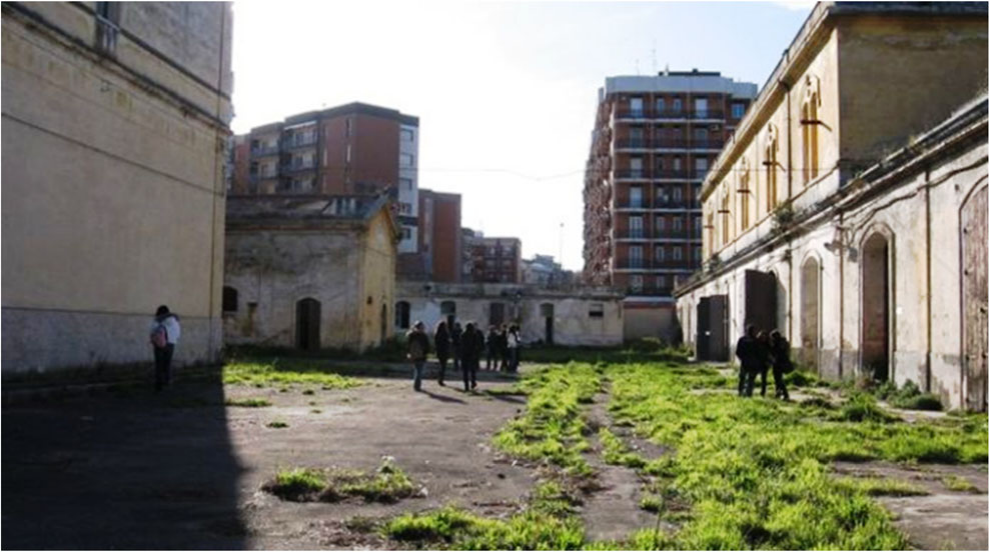
The military barracks “Rossani.” The houses built along the perimeter are visible

**Fig. 2:**
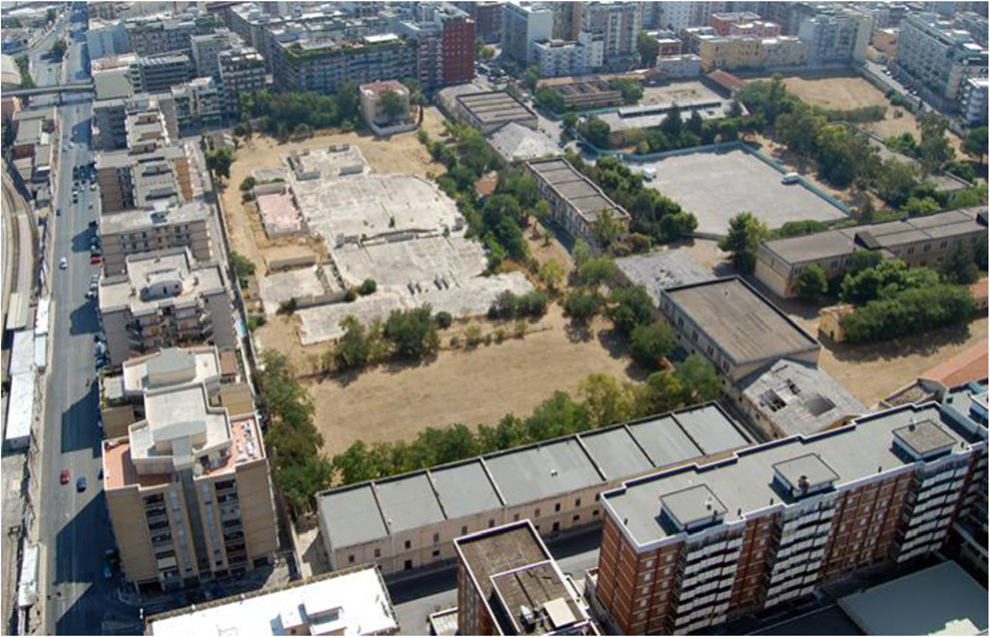
The military barracks “Rossani.” The houses built along the perimeter are visible

**Fig. 3:**
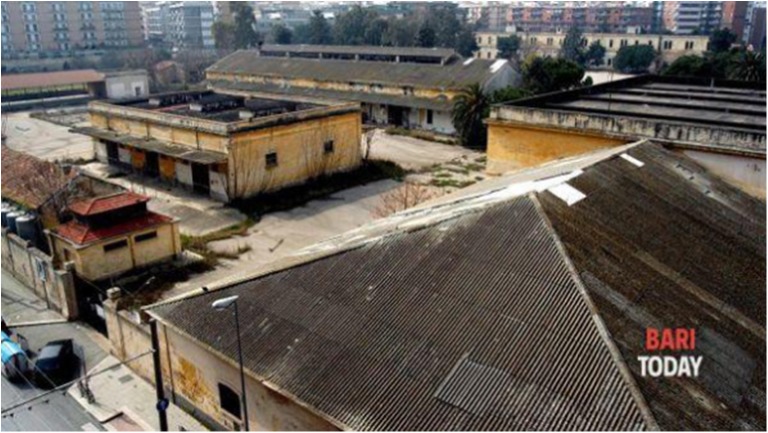
The military barracks “Rossani.” The houses built along the perimeter are visible

The military barracks, “Rossani,” were built between 1907 and 1912 (Figs. 1, 2, and 3). The use of materials such as asbestos, which was a cutting-edge material at that time, was huge, including all the roofs and the chimneys of the buildings. The barracks are located on a total area of 80,000 m^2^, with 14,000 m^2^ of covered space. In 1920, a transfer to the artillery barracks area “S. Lorenzo” occurred. In 1927, it was named “Rossani” barracks. In 1933, the Ministry of War decided to expand and set up the barracks “Duca delle Puglie,” which included the extension and elevation of the former carriageway, the construction of new stables for horses, replacement work on the old Eternit shells of the barracks, various improvement work, completion of the sewerage drainage network, and courtyard accommodation. The handrails at the barracks have Eternit covering the wooden beams and the Eternit coverings required continuous maintenance due to deterioration. During the earliest years, from 1920, the barracks were located outside the urban area. Later, in the 1930s and 1940s, urban expansion progressively reduced the distance from the closest residential areas to less than 5 m in a high-density demographic area. Between 1998 and 2000, dismantling of the barracks began.

In 2001, reclamation of the asbestos in the area began. From an initial estimate, 5000 m^2^ of roofs and tiles made of Eternit are needed to be removed. The first efforts intended to remove hazardous materials. In 2004, an inspection by Arpa Puglia, the Apulia environmental protection agency, certified that the work was not in accordance with the security regulations. The residues of the removed materials remained on the ground, and on windy days, the fibers were dispersed in the air. After the Arpa Puglia inspection, the job was stopped. In 2005, the work resumed despite strong protests by the citizens who were probably aware of the severity of this environmental emergency. During the wave of the “Fibronit problem,” few security measures and precautions were implemented at the work site. Specifically, the temporary deposition of hazardous waste, rather than transporting it immediately to a specialized landfill, was criticized. Following a new inspection by Arpa Puglia, the job was stopped because the most basic security standards were not adhered to. The public prosecutor suspended the activity of the firm that had won the contract. Non-compliance was related to fragments containing asbestos that were poorly guarded, a lack of decontamination units, and higher pollutant emissions than expected in the operational program. In 2006, the reclamation works were resumed and completed at the end of the year.

We report three peculiar cases of PMM that were caused by environmental (neighborhood) asbestos exposure close to the military barracks located in the urban area of Bari municipality. Additionally, we analyze the environmental exposure in the city, a contaminated town that is already classified as a site of national interest for land reclamation.

## Material and methods

### The Apulia mesothelioma registry

The National Mesothelioma Register (ReNaM) is active in Italy, and the notification of new cases is compulsory by law (DPCM n. 308/2002). It is organized in regional operational units that are responsible for data collection and exposure reconstruction and are coordinated by ReNaM, home of the national database.

The records of the three MM cases described here were listed in the Apulia regional mesothelioma register, the regional operational unit of the national registry. The register ensures complete, quality information on exposure and diagnosis due to the adoption of operative guidelines that have been agreed upon at the national level (Nesti et al. 2003). All cases in the register are from subjects with histologically confirmed mesothelioma. Histories of any occupational, i.e., certain, probable, or possible occupational exposure, or non-occupational, i.e., household, neighborhood, or environmental, exposure to asbestos are assessed and classified through face-to-face interviews conducted by trained interviewers with the subjects affected by mesothelioma or their relatives using a standardized questionnaire following ReNaM guidelines. The questionnaire is designed to determine demographic characteristics, lifestyle habits and, among others, lifelong occupational, military, and residential histories. The complete residential history, corresponding to the residence addresses throughout the life of an individual, includes the date of taking up and leaving the residence at each address. Information on the residential locations includes the house type, the address, and a description of each dwelling and its neighborhood environment, including the presence of close industries (asbestos cement, petro-chemical, railroad, shipbuilding industries, etc.). The questionnaire enables the collection of information on other circumstances of exposure to asbestos, such as the presence of asbestos-containing materials at home, asbestos cement tiles or water tanks, and the occupations of the persons with whom the patients lived with. In particular, the patient is asked to provide information on the longest occupation of each cohabitant (industry, job), how many years the patient lived with the cohabitant, whether the cohabitant used to bring dirty work clothes home, and whether the patient used to brush or wash them. Furthermore, exposure during hobby/leisure activities, starting from childhood, and school activities is investigated. Domestic activities that involve potential asbestos exposure are also investigated, including ironing on asbestos-coated ironing boards, do-it-yourself (DIY) projects in home maintenance and renovations such as small repair work (such as a masonry, plumbing, motor mechanics, or electrical work), thermal insulation work, the use of asbestos gloves, talcum powder use for personal hygiene, and the use of any asbestos-containing objects.

The patients were enrolled in the Apulia Regional Mesothelioma Register by the local occupational medicine unit. According to the standardized register procedures, we administered a questionnaire to investigate his lifestyle habits and work history, including any possible asbestos exposure during military service (Nesti et al. 2003).

## Results

### The histological, clinical, and genetic features and exposure of the cases

#### Case 1

In August 2015, a 74-year-old man suffering from familial Becker-type muscular dystrophy was admitted to the chest surgery division of “Ospedale San Paolo” in Bari, Apulia region, southern Italy, for dyspnoea and pleural effusion. Thoracentesis and intrapleuric coalescence were performed. His past medical history was unremarkable. He had been a smoker (7 cigarettes per day) since he was 20 years old. On the CT scan, extensive irregular thickening of the parietal and visceral pleura in the left hemithorax and mediastinal adenopathies were observed. Routine laboratory tests were unremarkable. A pleural biopsy was performed. Histology showed MM with a predominant solid epithelial pattern. The immunohistochemical analysis showed the positive expression of cytokeratins, calretinin, WT-1, and vimentin. TTF-1 was negative. The patient opted for chemotherapy (Alimta + cisplatin) with external hyperthermia. In January 2016, he underwent a seventh cycle of chemotherapy. In February 2016, he started radiotherapy. A total body PET scan showed increased pleural thickening and pleural effusion located in the left side. In September 2016, he underwent repeated chemotherapy cycles (Alimta + cisplatin) with external hyperthermia.

Regarding the patient exposure, he was exempted from military service due to pathology. The work histories of cohabiting family members and his family history of cancer were also evaluated. Exposure during leisure activities, travel, or hobbies and any exposure to ionizing radiation were excluded. The ascertained asbestos exposure was residential: he had lived near a source of asbestos pollution, asbestos in situ (in buildings), at a distance of less than 45 m away for 17 years from the age of 28 years. The subject had lived in an apartment overlooking the military barracks from 1969 to 1986.

At a follow-up in October 2017, more than 2 years after diagnosis and 27 months from the beginning of chemotherapy treatment, the patient had left shoulder pain and general weakness but good respiratory expansion.

#### Case 2

In September 2009, a 56-year-old woman was admitted to the chest surgery division of Hospital Bari Consorziale Policlinico for recurrent pleural effusion. Thoracentesis and intrapleuric coalescence were performed. Her past medical history reported hysterectomy in 1999 and myocardial infarction in 2008. She had been a smoker (20 cigarettes per day) since she was 20 years old until 2008. On the CT scan, extensive irregular thickening of the parietal and visceral pleura in the left hemithorax was observed. A pleural biopsy was performed. Histology showed MM with a predominant solid epithelial pattern. The immunohistochemical analysis showed the positive expression of cytokeratins, calretinin, WT-1, and vimentin. TTF-1 was negative. Ki67 was 5%. The patient opted for surgery at a specialist center in Brescia, Italy, and was subjected to left pleuropneumectomy, followed by cycles of chemotherapy (Alimta + cisplatin). She died after 28 months in 2011.

Regarding the patient exposure, the work histories of cohabiting family members and her family history of cancer were also evaluated. Exposure during leisure activities, travel, or hobbies and any exposure to ionizing radiation were excluded. The ascertained asbestos exposure was environmental due to both her workplace, she was a bank employee from 1986 to 1996 in an agency 50 m far from Rossani barracks, and her residential history, she had lived near the same source of asbestos pollution, asbestos in situ (in buildings), at a distance between 50 and 500 m for 30 years from the age of 26 years, from 1979 to 2009.

#### Case 3

In February 2013, a 54-year-old woman was admitted to the chest surgery division of Hospital La Madonnina Bari for recurrent pleural effusion. Her past medical history was unremarkable. She had been a smoker (10 cigarettes per day) since she was 29 years old until 2013. On the CT scan, irregular thickening of the parietal and visceral pleura in the right hemithorax was observed. Thoracoscopy and histological examination diagnosed MM with a solid epithelial pattern. The immunohistochemical analysis showed the positive expression of cytokeratins, calretinin, CEA, WT-1, and vimentin. The patient opted for surgery at a specialist center in Brescia, Italy, and was subjected to right pleuropneumectomy, followed by cycles of chemotherapy (Alimta + CDDP).

Regarding the patient exposure, the work histories of cohabiting family members and her family history of cancer were also evaluated. Exposure during leisure activities, travel, or hobbies and any exposure to ionizing radiation were excluded. The ascertained asbestos exposure was environmental due to both her workplace, she was employed in regional offices from 1980 to 2000 100 to 250 m far from the Rossani barracks, and her residential history, she had lived near the Fibronit site at a distance of 200 m for 54 years since birth from 1959 to 2013.

As a follow-up in October 2017, more than 4 years (55 months) after treatment, the patient is alive and disease-free without recurrence.

## Discussion

### Environmental asbestos exposure

Since 1960, in the first epidemiologic study in South Africa, a risk of pleural mesothelioma was shown to be associated with asbestos exposure; some of the cases reported were attributed to environmental exposure (Wagner et al. 1960) The MM cases presented here are emblematic cases of environmental exposure in a large polluted city. In fact, the general population often suffers from exposure to environmental contaminants that cannot be directly controlled by the individual. In a recent review, Liu et al. (2017) summarized the most recent studies of the association between MM and environmental asbestos exposure. EE is defined as neighborhood exposure based on residence in close proximity to industrial/mining sources of asbestos or residence in urban or polluted areas. EE is also defined as any exposure that occurs during residence in a town where asbestos-processing plants were located. By the way, IARC 2012, no. 100 reported that “In studies of asbestos concentrations in outdoor air, chrysotile is the predominant fibre detected. Low levels of asbestos have been measured in outdoor air in rural locations (typical concentration, 10 fibres/m^3^ [f/m^3^]). Typical concentrations are about 10-fold higher in urban locations and about 1000 times higher in close proximity to industrial sources of exposure(e.g. asbestos mine or factory, demolition site, or improperly protected asbestos-containing waste site).”

When mesothelioma is due to environmental exposure, the M:F sex ratio is 1:1 and the median age at diagnosis is 60 years: in the cases mentioned here, it is reported to be 61 years. Asbestos is an environmental carcinogen, and asbestos-related diseases are a global-scale public health issue. The cumulative exposure to asbestos and duration of exposure increase the MM risk (Espina et al. 2015; Imai and Hino 2015). The role of non-occupational asbestos exposure (para-occupational, domestic, or environmental) in the occurrence of MM has already been demonstrated in several studies (Bourdès et al. 2000; Magnani et al. 2001; Pasetto et al. 2005; Ferrante et al. 2007; Goldberg and Luce 2009; Lacourt et al. 2014). In a recent review and meta-analysis, Marsh et al. (2017) confirmed an increased risk of pleural MM from non-occupational (neighborhood) asbestos exposure (RR= 6.9; 95% CI 4.2 to 11.4).The main feature of non-professional exposure is the long latency and duration of exposure in subjects often very young at the beginning of exposure. Also in analysis limited to non-occupationally exposed subjects, the risk of MM increases with cumulative doses of asbestos.

In our cases (Table 1), exposure started at birth or at 26–28 years of age, with a duration of 17 to 54 years and latency between 30 and 54 years.

**Table 1:** The exposure features of the cases

Exposure features	Case 1	Case 2	Case 3
Sex	Male	Female	Female
Anatomical site	Pleura	Pleura	Pleura
Year of diagnosis	2015	2009	2013
Age at diagnosis (years)	74	56	54
Year of first exposure	1969	1979	1959
Age at first exposure (years)	28	26	1
Hours/week	168	168	168
Frequency	Continuous	Continuous	Continuous
Duration of exposure (years)	17	30	54
Calendar years of exposure	1969–1986	1979–2009	1959–2013
Latency (years)	46	30	54
Distance of the home or workplace from the source of pollution: military barracks (Rossani) (meters)	45 homes	50 workplaces, 500 homes	100 workplaces
Distance of the home from: asbestos cement factory (Fibronit) (meters)	1100	1200	200

The duration of exposure is the duration of the residence period; it is a proxy of the cumulative dose to which the residents are exposed, that is, an estimate of effective exposure (Magnani et al. 2015).

Other studies have detected a significant MM risk caused by residential proximity to asbestos cement plants in the absence of occupational exposure (Fazzo et al. 2014; Mensi et al. 2015). These studies highlight the importance of assessing the impact of asbestos exposure not only among workers but also among their cohabitating family members and in the general population.

In Italy, several of these areas have been included among national priority contaminated sites and environmental remediation has been prescribed, but in Bari, it has only been partially implemented (Pirastu et al. 2013).

Orenstein and Schenker (2000) studied the associations between the residential distance from environmental asbestos, decreased exposure duration, and the MM risk. Studies of neighborhood exposure have reported that increased distance from the sources is associated with a decreased MM risk (Maule et al. 2007; Musti et al. 2009). Moreover, it is known that worn asbestos products can release asbestos fibers which have the same carcinogenic potency as “standard” chrysotile (Spurny 1989).

Our patients (Table 1) had lived or worked at distances 45 to 100 m from the source of pollution considered, the military barracks. Regarding the Fibronit site, all patients had lived from 200 to 1200 m far. In particular, the case number 3 have had an important exposure because she lived and worked closely the two pollution sources. With these features, the typical time variable for environmental exposure of the cases here discussed may be considered as continuous for 168 h per week. By the way, the latest data published by ReNaM, the Italian national mesothelioma registry, referred to 21.463 cases occurred in the period 1993–2012, showed 694 cases with ascertained environmental exposure, 4.2% of total cases reported (Quinto rapporto 2015).

The study of MM due to the environmental risk is often hindered because of the long latency period and the small number of cases. Additionally, this type of exposure is involuntary and is unknown in most cases (Baumann and Carbone 2016). In Italy, the ReNaM data (Marinaccio et al. 2015) show that people with EE, in 514 cases from 1993 to 2008, were younger at diagnosis (67.2 versus 68.1 years [*p* = 0.01]), were younger at first exposure (18.5 versus 22.5 years [p = 0.0001]), and had longer latency times (49 ± 14 versus 46 ± 12 years) with respect to occupationally exposed patients with MM. Consistent with these data, our patients (Table 1) were diagnosed at ages from 54 to 74 years and were younger at first exposure, with latencies from 30 to 54 years.

### Sources of environmental exposure in the town: the asbestos cement factory (Fibronit) and military barracks (Rossani)

**Fig. 4:**
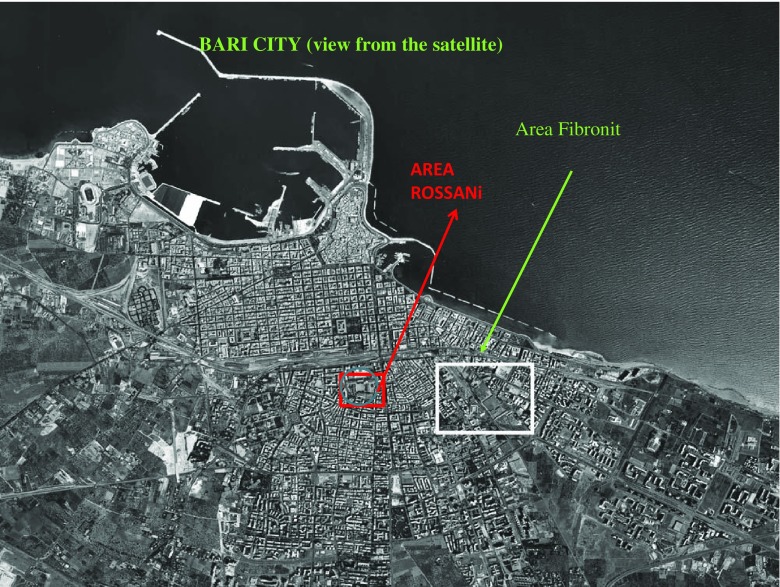
Bari map: Fibronit and Rossani sites

This study extends our previous investigations (Bilancia et al. 2003; Musti et al. 2009) and, consistent with the literature, confirms the adverse health effects of asbestos environmental pollution in the town of Bari. The distance between the two contaminated sites described here is 1400 m (Fig. 4).

Continuous airborne emissions were due to dispersion from the barracks area and from the asbestos cement factory area by wind. Data on the environmental asbestos concentration inside the barracks area and the Fibronit site were not available. The concentration of asbestos fibers in the ambient air close to asbestos cement factories in Bari has been inadequately monitored in the past, when the factory was active. Fiber concentration measurements related to Fibronit were only available with limited data for the latest years. Until 1970, up to 20 fibers/cm^3^ of airborne asbestos (length 5 μm and diameter 0.3 μm) were measured. Measurements in the following years were reported up to 10 fibers/cm^3^ in 1972 and from 4 to 19 fibers/cm^3^ in 1974 after reclamation (Coviello et al. 2002). However, the relevance of the environmental exposure was not considered until the mid-1970s, when environmental measurements were taken close to the plant in nearby areas located far from the roads, without much pollution from urban traffic. Mean concentration values equal to 16.06 × 10^−4^ “particles (5 micron in size) per cc. of air” were reported (Napoli 1975) but are certainly not representative of the high pollution level in earlier decades. The amount of environmental pollution from asbestos present at the time in the city of Bari, corresponding to the years of exposure in the cases discussed here, is also given by a comparison with the current level of background pollution. The reference background level exposure index corresponds to a mean lifetime cumulative exposure of “less than 0.1 fibres/mL-y, an average concentration of approximately 0.1 fibres/l”, as reported by the International Agency for Research on Cancer (IARC) monograph no. 100 (IARC International Agency for Research on Cancer (IARC) 2012). Moreover, WHO estimated that “with continuous exposure to 0.4–1 fibre/l, the lifetime risk of MM would be from (4 to 10) × 100,000. Linear extrapolation to 0.1 fibre/l (the current background level) would correspond to lifelong excess in the order of one case (from 0.4 to 2.5) of MM in every 100,000 persons” (World Health Organization Regional Office for Europe 2000).

The diffusion process of the asbestos fibers, both from the factory and from the military barracks to the surrounding zones, was favored by physical mechanisms and has been confirmed by the history of urban expansion in the city of Bari around the two sites. Also, the deterioration due to time and weather has contributed to the release of asbestos fibers into the city environment.

It is known that the concentration of asbestos fibers in the air surrounding the emission point depends on wind direction and velocity (Laamane et al. 1965). Abakay et al. (2016) studied the risk of developing environmental mesothelioma for inhabitants near naturally occurring asbestos (NOA) source. They found that the distance of a residence from NOA and the predominant wind direction can influence the MM risk. Also, Kurumatani and Kumagai (2008) and Tarrés et al. (2013) studied the effect of meteorological conditions on MM. Also in their studies, the dominant wind direction influenced the MM risk. Especially, Kurumatani and Kumagai (2008) suggest that a parameter that includes meteorological conditions can be a better proxy for exposure assessment than residential distance alone, in the study of the effects of environmental exposure to asbestos in populations living in polluted areas.

Fazzo et al. (2016), in a study on the incidence of cancer, reported that the highest values around polluting industries were consistent with prevailing wind directions and confirmed that the air quality of CS areas is affected by industrial atmospheric emissions.

In this study, meteorological data from the period of interest, from 1912 for Rossani and from 1933 for Fibronit, were not available, so the meteorological data from more recent years, namely, 1961–1990, were considered in our previous study (Musti et al. 2009). The direction of the winds was also evaluated as a possible cause of exposure to verify the predominant direction and the relative geographical distribution of MM cases. Data on the frequency of wind by intensity and direction were acquired from the “Bari Palese Macchie Station” of the Military Air Force National Center for Meteorology and Aeronautical Climatology. The station provided the monthly ground frequency distributions for each of the synoptic hours (00, 03, 06, 09, 12, 15, 18, 21). The period covered by the climatic processing is from 1961 to 1990, approximately the same years of exposure in our three cases. The analysis highlighted the distribution of the wind (the average over the period 1961–1990) during the various months of the year. Wind diffusion did not seem to play an important role given the absence of clearly predominant winds. So it corroborates with the hypothesis that the proximity to the source of risk may increase the incidence of disease (Musti et al. 2009).

Moreover, our previous study (Barbieri et al. 2012) of asbestos fiber burden in the lungs of eight pleural mesothelioma patients residing near asbestos cement plants in Piedmont and Apulia regions who were not occupationally exposed to asbestos showed values ranging from 110,000 to 4,300,000 fibers per gram (f/g) of dry lung. Five patients, with ages at diagnosis from 36 to 65 years, lived at distances ranging from 200 to 2000 m from the Bari Fibronit plant between 1960 and 1997.

Based on the information on the asbestos types, we found, in that study, a detectable amount of mainly amphibole asbestos fibers, and the long persistence of these fibers could represent a marker of EE. In particular, in three non-occupationally exposed MM patients, there were 110,000 (resident at a distance of 2000 m) and 1,700,000 (resident at a distance of 200 m) ff/g of dry lung in two men and 2,300,000 ff/g of dry lung in a woman (resident at a distance of 500 m). These data confirm a direct relationship between the lung fiber burden and the distance between the residence and the factory (Barbieri et al. 2012).

Due to these data on asbestos pollution in Bari, we can hypothesize the past asbestos exposure of the three new cases here discussed that lived at a distance from 200 to 1200 from Fibronit.

The second Italian Consensus Conference on Malignant Mesothelioma of the Pleura has confirmed that the quantitative relationship between MM and asbestos exposure increase with cumulative exposure to asbestos, the lung fiber burden, and the duration of exposure (Pinto et al. 2013). In the study of MM epidemiology, cumulative exposure is a proxy for relevant exposure and the duration and the intensity of exposure are independent determinants of MM occurrence (Pinto et al. 2013). The same conclusion was reported in the third Italian Consensus Conference on Malignant Mesothelioma of the Pleura (Magnani et al. 2015; Novello et al. 2016). Furthermore, a recent case-control study (Ferrante et al. 2016) explored the relationship between cumulative exposure and pleural MM after non-occupational exposure and investigated the risk associated with asbestos materials in residential areas, with a cumulative exposure index to estimate the frequency, duration, and intensity of exposure. The study showed a relationship between the pleural MM risk and cumulative exposure after non-occupational exposure and confirmed the quantitative relationship between the MM incidence and cumulative exposure to asbestos, even at low levels of exposure. Consistent with Ferrante et al. (2016), in the present study, the assessment of EE was based on the distance between the home and the pollution source.

The national register of mesothelioma (ReNaM) documented that 10.2% of MM cases are due to non-occupational exposure to asbestos (Marinaccio et al. 2015). In particular, in our regional register, 10.9% of cases are due to environmental exposure (Quinto rapporto 2015). These data confirm the difficulty in recognizing and attributing non-occupational exposure to asbestos even though this type of exposure is becoming increasingly more common among new cases of mesothelioma. Consistent with Armstrong and Driscoll (2016), this finding can be defined as the “third-wave exposure.” Indeed, they defined “third-wave exposure as both occupational and non-occupational exposure to asbestos as a consequence of repairs, renovations, demolition of buildings and environmental exposure to asbestos”.

The history of these military barracks shows that the deterioration of asbestos in situ, the removal of asbestos, and the related exposure require accurate monitoring of asbestos fiber concentrations in urban air and in areas proximal to circumstances that are thought to present a particular hazard, such as the renovation or demolition of homes and buildings constructed with asbestos cement products. The current scientific knowledge was not known in the 1960s and 1970s, when the Rossani barracks were used. In all the cases reported here, we assessed EE via the lifetime residential distance from the sources of environmental exposure in the town, the asbestos cement factory (Fibronit) and military barracks (Rossani), the calendar years of residence, and the duration of residence (Table 1) as proxies for the intensity of exposure. Finally, to better assess EE to asbestos in addition to collecting information via direct, face-to-face interviews, we performed a historical reconstruction of asbestos pollution in the two sources within the urban perimeter using residential histories and the periods 1969–1986, 1979–2009, and 1959–2013, when respectively the three subjects were exposed. This kind of investigation is consistent with the World Health Organization’s comprehensive approach in the assessment of the health status of residents in contaminated sites (WHO 2013) and with recommendations for epidemiological surveillance programs (Zona et al. 2014). The public health relevance of environmental asbestos exposure in Italian national priority contaminated sites, such as Bari, has also been stressed in the final report of the governmental conference on asbestos and ARDs (Comba et al. 2013).

## Conclusion

It is confirmed, according to Binazzi et al. (2017), that asbestos environmental pollution is a risk for people living in contaminated sites. The past extensive use of asbestos has generated severe public health consequences among Bari inhabitants and these cases emphasize the association between MM and asbestos environmental pollution in the city. The presence of the AC factory and the military barracks has been correlated with the onset of malignant mesothelioma among the neighboring resident population. Asbestos pollution from both sources can greatly increase the mesothelioma risk. The diffusion process of the asbestos fibers from the military barracks to the surrounding zones was favored by physical mechanisms, which has been strongly confirmed by tracing the progressive urbanization phenomenon of the city of Bari around the barracks. In the 1930s when the barracks opened, the site was outside the urban area. Over the following three to four decades, the city grew and incorporated the barracks. The failure to reclaim and safely decontaminate the area inside the city has been a serious public health problem. Consistent with the second Governing Conference (November 2012) and the National Asbestos Plan (2013), the theme of MM cases of environmental origin was identified as a research priority with a specific mandate for ReNaM and COR: “regions will have to, by committing the COR Regional or other competent structures, investigate the magnitude of mesothelioma risk connected to non-professional exposure (environmental or para-occupational).” It must also be stressed that the scientific support of the case series collected by our regional mesothelioma register and the continuous documentation of the effects of environmental asbestos exposure have increased awareness among the citizens of Bari and have prompted the authorities to schedule the decontamination of the site to safeguard public health.
